# Engineering dendritic cell vaccines to improve cancer immunotherapy

**DOI:** 10.1038/s41467-019-13368-y

**Published:** 2019-11-27

**Authors:** Caleb R. Perez, Michele De Palma

**Affiliations:** Swiss Institute for Experimental Cancer Research (ISREC), School of Life Sciences, École Polytechnique Fédérale de Lausanne (EPFL), CH-1015 Lausanne, Switzerland

**Keywords:** Cancer immunotherapy, Tumour immunology

## Abstract

At the interface between the innate and adaptive immune system, dendritic cells (DCs) play key roles in tumour immunity and hold a hitherto unrealized potential for cancer immunotherapy. Here we review the role of distinct DC subsets in the tumour microenvironment, with special emphasis on conventional type 1 DCs. Integrating new knowledge of DC biology and advancements in cell engineering, we provide a blueprint for the rational design of optimized DC vaccines for personalized cancer medicine.

## Abstract (web summary)

Dendritic cells (DCs) have been explored as a promising strategy for cancer immunotherapy. In this Perspective, the authors discuss the different types of DCs and their therapeutic potential in the context of vaccines for personalized cancer therapy.

## Introduction

Reprogramming of the immune system against cancer has shown clinical promise in recent years. Dendritic cells (DCs) are a particularly interesting immunotherapeutic target given their ability to uptake and present tumour-associated antigens (TAAs) through a variety of mechanisms (Box 1), priming potent effector responses against the tumour^1–3^. Besides direct antigen presentation, additional DC-intrinsic properties are relevant for immunotherapy, including the capacity to migrate between lymphoid and non-lymphoid tissues and modulate cytokine and chemokine gradients to control inflammation and lymphocyte homing, all of which are likely important for systemic and long-lasting anti-tumour effects. To leverage these features, personalized vaccines comprising patient-derived DCs manipulated ex vivo have been extensively studied. These therapies are generally manufactured by isolating monocytes or hematopoietic stem and progenitor cells (HSPCs) from peripheral blood, which are subsequently treated with recombinant cytokines to induce differentiation, stimulated to induce maturation, and loaded with TAAs of various forms, a process that has been utilized in numerous preclinical and clinical studies^4^.

Although multiple clinical trials have demonstrated the safety and immunogenicity of DC vaccines, clinical responses have been largely disappointing. This can be attributed in part to functional deficiencies in the cells that comprise conventional vaccine formulations—such as insufficient antigen presentation, migratory capacity, and cytokine release—which poorly equip them to overcome the immunosuppressive tumour microenvironment that limits DC and effector cell function^4–6^. Recent studies have begun to expand our knowledge of the roles that DCs play in anti-tumour immune responses, and have revealed new methods for the generation of DC vaccines. In this Perspective, we lean on these investigations to identify types of DCs with the greatest therapeutic potential, as well as strategies to engineer them toward improved efficacy. Synthesizing this information, we aim to provide a blueprint for the design and manufacture of the next generation of personalized DC vaccines.

Box 1 Mechanisms of TAA presentation by DCs

- **Cross-presentation—**Presentation of internalized TAAs on endogenous MHCI, capable of inducing the activation of TAA-specific CD8^+^ T cells. Antigens are processed via two distinct intracellular pathways following internalization^100^: (i) the cytosolic pathway, in which antigens are exported to the cytosol from the endosome and processed by the proteasome into antigenic peptides that are loaded onto MHCI, and (ii) the vacuolar pathway, in which antigens are processed by lysosomal proteases and loaded onto MHCI directly in the endosome.
- **Cross-dressing**—Uptake and recycling of pre-formed peptide-MHC complexes shed by tumour cells, capable of inducing the activation of TAA-specific CD8^+^ T cells without the need for further processing. Transfer of peptide-MHC complexes is proposed to occur via trogocytosis^101^ (inter-cellular transfer of plasma membrane fragments) or transfer of EV^71,102^.
- **Antigen transfer—**Transfer of TAA peptides processed by exogenous cells to DCs and subsequently loaded on endogenous MHCI, capable of inducing the activation of TAA-specific CD8^+^ T cells. Transfer of antigenic peptides is proposed to occur through gap junctions^103,104^.
- **MHCII-restricted presentation**—Presentation of internalized TAAs on MHCII, capable of inducing the activation of TAA-specific CD4^+^ T cells^105^. Antigens are processed in endosomal compartments by lysosomal proteases to yield antigenic peptides, after which peptide-MHCII complexes are generated and transported to the plasma membrane for presentation.

## Contribution of DCs to anti-tumour immunity

DCs consist of multiple distinct subsets, most commonly classified by ontogeny, including four major populations; the full DC landscape has been reviewed elsewhere^1^. These include plasmacytoid DCs (pDCs); conventional DCs (cDCs), which are further split into type 1 (cDC1) and type 2 (cDC2); and monocyte-derived DCs (MoDC). Each subset differs in its capacity for antigen presentation, migration, and cytokine secretion. Thus, the choice of cell type is a crucial parameter in the engineering of DC vaccines.

### Monocyte-derived DCs

MoDCs comprise a heterogeneous population of antigen-presenting cells (APCs) that arise mainly from monocyte precursors in response to inflammation^1^. The majority of clinical trials utilize MoDCs generated ex vivo for operational reasons, as large populations of mature antigen-loaded MoDCs can be easily obtained from peripheral blood-derived CD14^+^ monocytes or CD34^+^ HSPCs via treatment with granulocyte-macrophage colony-stimulating factor (GM-CSF) and interleukin 4 (IL-4)^4,6^. MoDCs generated ex vivo have been tested extensively in vaccination studies^4,6,7^, which demonstrated their capacity to cross-prime T cells^8–10^ and produce anti-tumoural cytokines, such as IL-12 (ref. ^11^). This has translated to anti-tumoural activity in a subset of treated patients, illustrating the potential of MoDCs as a valuable vaccine consitutent^11,12^. However, the magnitude of clinical response has been modest in the majority of patients, perhaps owing to incomplete recapitulation of physiological MoDC development captured by ex vivo differentiation methods. Indeed, GM-CSF and IL-4 treatment of both murine and human precursors has been shown to produce MoDCs that are transcriptionally and phenotypically distinct from their naturally occurring (primary) counterparts^10,13^. This distinction appears to translate to functional deficiencies in ex vivo-derived MoDCs, such as reduced T cell priming capacity relative to CD11c^+^ DCs isolated from peripheral blood^14^. In addition, MoDCs generated ex vivo may have limited migratory capacity to lymph nodes^15–17^, likely contributing to the suboptimal efficacy of such vaccine formulations.

### Conventional type 1 DCs

Mounting evidence suggests that cDC1s play an integral role in tumour immunity and represent a promising alternate cell type for vaccination purposes. Bulk gene expression profiles of whole tumours indicate that cDC1 gene signatures correlate with improved prognosis across a wide range of malignancies, although inconsistency in what constitutes cDC1-specific gene signatures could confound this analysis^3^. Recently published single-cell RNA sequencing (scRNA-seq) analysis of non-small-cell lung cancer (NSCLC) samples, in which immune cells were grouped by unsupervised clustering without the bias of pre-selected reference genes, provided strong evidence that cDC clusters correlate with improved survival^18^. Numerous pre-clinical studies that used cDC1 depletion models based on mice deficient in *Batf3* (also known as basic leucine zipper ATF-like transcription factor 3), a transcription factor crucial for cDC1 development^19^, demonstrate the requirement of cDC1s for immunogenic tumour rejection^20,21^, as well as for responses to immunotherapies such as immune checkpoint blockade^22,23^ and adoptive T cell transfer^24^.

There appear to be multiple mechanisms by which cDC1s mediate tumour immunity, involving a range of specialized functions. Perhaps most importantly, cDC1s are known to be highly efficient in the cross-presentation on major histocompatibility class I (MHCI) molecules of exogenous antigens (Box 1) to CD8^+^ T cells^1^, a cell type strongly correlated with prognosis across various cancers^25^. Indeed, cross-presentation specifically by cDC1s was shown to be necessary for tumour rejection^20^. The BEACH domain-containing protein WDFY4 was identified by clustered regularly interspaced short palindromic repeats (CRISPR) screen to be necessary for cross-presentation in both primary splenic cDC1s and *ex vivo*-differentiated cDC1s, and *Wdfy4*^*–/–*^ mice failed to mediate rejection of a highly immunogenic fibrosarcoma model. Although exact mechanisms are not yet clear, it was proposed that WDFY4 regulates vesicular trafficking pathways important to antigen processing. Of note, loss-of-function was both cell-type specific, having no impact on the presentation of TAAs by MoDCs or cDC2s, and was limited to cross-presentation alone, as it did not compromise cDC1 development, cytokine secretion, or antigen presentation on MHCII. This indicates that cross-presentation, specifically by cDC1s, was indispensable for tumour rejection in that fibrosarcoma model. These results are consistent with previous studies reporting distinct cross-presentation mechanisms between cDC1s and other DC subsets^8^.

Multiple studies have shown that the contribution of cDC1s to the anti-tumour response extends beyond cross-presentation. Rescuing cDC1 development in *Batf3*^*–/–*^ mice by expressing an interferon regulatory factor 8 (*Irf8*^*VENUS*^) transgene—a transcription factor required for cDC1 lineage commitment that is normally maintained by BATF3-mediated autoactivation^19^—yields cDC1s with the ability to cross-present as normal, yet fail to reject the fibrosarcomas^21^. Although this implicates some BATF3-dependent function in cDC1s that is important for tumour rejection but independent of cross-presentation, the exact mechanisms are not fully understood. One likely pathway involves the modulation of immune cell migration by cDC1s, driven by a variety of chemokine-receptor interactions that recruit different immune cells both into the tumour and toward lymph nodes. For example, cDC1-derived chemokine (C-X-C motif) ligand 9 (CXCL9) and 10 were shown to promote C-X-C chemokine receptor 3 (CXCR3)^+^ T cell infiltration into the tumour^24^, whereas XCR1^+^ cDC1s were shown to migrate toward tumour-derived XCL1^26^. The presence of these chemokines in the tumour microenvironment was necessary for cDC1-dependent anti-tumour responses across a variety of tumour models^22,24,26,27^, hinting at the importance of cDC1s to both secrete and respond to tumour-derived chemokines. Expression of C-C chemokine receptor 7 (CCR7) by DCs is important for their trafficking to tumour-draining lymph nodes^28–30^. In melanoma models, CCR7 expression in cDC1s was necessary for the trafficking of TAAs toward lymph nodes, where effector T cell priming occurred and triggered anti-tumour responses^28,30^. Together, these studies highlight the importance for cDC1s to both infiltrate the tumour, allowing recruitment of other DCs and lymphocytes via chemokine secretion, and migrate to lymph nodes, allowing presentation of TAAs to effector T cells.

Two recent studies identify cDC1-secreted cytokines as determinants for the efficacy of immune checkpoint blockade in MC38 models of colon adenocarcinoma^22,31^. In one study, a combination of scRNA-seq and intravital live cell imaging showed that IL-12 was secreted by cDC1-like cells in response to interferon-γ (IFN-γ) produced by CD8^+^ T cells bound to an anti- programmed cell death protein-1 (PD-1) antibody; this crosstalk was necessary for re-activation of exhausted T cells and subsequent anti-tumour responses^31^. cDC1-derived CXCL9 was similarly identified as necessary for tumour regression on PD-1 blockade^22^. Interestingly, CXCL9 acted only to drive CD8^+^ T cell proliferation and re-activation, as opposed to its canonical function as a T cell chemoattractant. This is consistent with previous reports using an alternative checkpoint blockade therapy, anti-TIM-3 (also known as T-cell immunoglobulin and mucin-domain containing-3) (ref. ^27^). CXCL10, another chemoattractant that engages the same receptor (CXCR3) and is secreted preferentially by cDC2s, did not modulate tumour response to checkpoint blockade^22^. Together, these independent studies point to a pivotal role of cDC1s in mediating immune activation via cytokine secretion.

In summary, numerous studies suggest that cDC1s are potent initiators of the immune response against cancer, engaging multiple facets of the immune system. Importantly, methods have also been developed to generate ex vivo cDC1s that resemble their primary counterparts, suggesting that the anti-tumoural functions of cDC1 might extend to cDC1s generated ex vivo for vaccine formulations (see below). By leveraging the abilities of cDC1s to (i) potently cross-prime CD8^+^ T cells, (ii) manipulate chemokine gradients to control immune cell infiltration into the tumour, (iii) deliver TAAs to lymph nodes, and (iv) secrete cytokines that modulate tumour-driven immunosuppression, cDC1s hold significant promise for improving DC-based tumour vaccines.

### Conventional type 2 DCs

Other DC subsets have also demonstrated immunotherapeutic relevance, although the breadth of evidence is not as expansive as for MoDCs and cDC1s. cDC2s specialize in priming CD4^+^ T cells via antigen presentation on MHCII (Box 1), and are thus capable of efficiently polarizing tumour-infiltrating lymphocytes (TILs) toward anti-tumour T helper 1 (Th1) or Th17 phenotypes^1,2^. Human cDC2s have also demonstrated the capacity to cross-present antigen to CD8^+^ T cells and secrete IL-12 under certain conditions^32–34^. By scRNA-seq, intra-tumoural levels of cDC2s correlated positively with the survival of patients with NSCLC^18^.

cDC2s were shown to drive CD4^+^ anti-tumour immunity in a B16-F10 melanoma model after depletion of regulatory T cells (T_regs_), which suppress cDC2 migration and function^35^. cDC1s, presumably not actively suppressed by T_regs_ in this model, were dispensable for the anti-tumoural effects of T_reg_ depletion. In contrast, in the context of PD-1 blockade in the same tumour model, cDC1-derived IL-12 was necessary for an anti-tumour response^31^, suggesting distinct, potentially synergistic, therapeutic pathways mediated by different cDC subsets. As another example, a vaccination study demonstrated the ability of tumour-derived cDC2s to induce Th17 polarization of CD4^+^ T cells, causing the downstream reprogramming of tumour-associated macrophages (TAMs) from a pro-tumoural M2-like phenotype to an anti-tumoural M1-like phenotype^36^. This induced a strong anti-tumour response in a lung carcinoma model characterized by high TAM infiltration. However, the magnitude of this effect was smaller in a B16 model with low TAM infiltration, in which cDC1s exhibited a greater anti-tumour response, presumably driven by stronger CD8^+^ T cell priming, in the absence of TAMs. On the basis of the anti-tumoural functions observed in some tumour models, primary cDC2s isolated from human blood have been clinically tested in vaccination trials against melanoma, demonstrating immunogenicity and survival benefits in some patients^37^.

### Plasmacytoid DCs

pDCs are known for the production of large amounts of type-I IFNs in response to viral infections^2,38^. On the one hand, pDCs have been implicated in cancer-associated immunosuppression, mainly through stimulation of pro-tumoural T_regs_ via inducible costimulatory ligand (ICOS-L) secretion^39–41^. This role likely explains the correlation between intra-tumoural pDC levels and tumour progression across multiple malignancies^39–41^. On the other hand, pDCs have also demonstrated importance in the initial response to cancer vaccines^42^. After treatment with a nanoparticle-encapsulated RNA vaccine, pDC-derived type-I IFNs were shown to be necessary to mature cDC1s, which in turn primed effector anti-tumour responses, again suggesting a possible benefit of engaging multiple DC subsets. Indeed, the benefit of pDC-cDC1 crosstalk has been documented in an anti-viral context^43^. In addition to type-I IFN production, primary pDCs have also shown a capacity for T cell cross-priming and IL-12 release^34^. Accordingly, pDCs isolated from the human blood induced IFN-driven T cell responses in melanoma patients^44^. Although concerns for pDC-mediated immunosuppression may exist^39–41^, the aforementioned pre-clinical and clinical results suggest that pDCs may hold promise as effective anti-cancer vaccine constituents.

### Multiplexed DC vaccines

Undoubtedly, all DC subsets likely contribute to generating anti-tumour responses, with the relative contribution of each depending on the type of malignancy—as demonstrated by the model-dependence of cDC2-based vaccines^36^—and treatment strategy. It is tempting to speculate that an ideal vaccine would package multiple DC subsets to take advantage of their complementary functions and crosstalk between cell types. Further investigations are warranted to better elucidate the specific roles that each DC subset plays in anti-tumour immunity; such knowledge may indeed guide the development of multiplexed DC vaccines. In addition, advancements in ex vivo differentiation methods are needed to allow the generation of DC subsets with intact immunogenic capacity.

In the following sections of this Perspective, we will focus on methodology and strategies for generating DCs, especially cDC1s, with potentially improved performance in cancer vaccination applications.

## Vaccine manufacturing: optimizing ex vivo differentiation

Although cDC1s have shown promise as a vaccine candidate, to our knowledge, no clinical trials currently utilize ex vivo-derived cDC1s for adoptive cell transfer. This is mainly due to difficulties in generating cDC1-like cells at a scale amenable to therapeutic applications, while maintaining the phenotype and function of bona fide cDC1s. The incorporation of FMS-like tyrosine kinase 3 ligand (FLT3L), a cytokine known to drive physiological development of cDCs and pDCs, into murine bone marrow (BM)^45,46^ or human HSPC^13,47,48^ cultures yields a mixture of cells that resemble bona fide pDCs, cDC1s, and cDC2s. However, cDC1 enrichment and yield are generally poor in both murine and human cultures, with the former largely containing cDC1-like cells that lack expression of several markers of primary murine cDC1, such as CD8α and DEC205^45,46,49^. Recent investigations, outlined below, have attempted to address these limitations, revealing new methods that could improve the feasibility of cDC1-based vaccines.

### Cell culture conditions and cytokine cocktails

The Notch signalling pathway plays a crucial role in differentiation of many cell types, including DCs^50,51^. Two independent studies recently demonstrated the importance of Notch signalling in recapitulating cDC1 differentiation ex vivo, leveraging murine OP9 stromal cells expressing the Notch ligand delta-like 1 (OP9-DL1) to improve the yield and phenotype of ex vivo-differentiated cDC1s^49,52^. Transfer of murine BM precursors to OP9-DL1 monolayers after 3 days of normal FLT3L treatment resulted in cells expressing bona fide murine cDC1 markers—namely CD103, CD24, DEC205, and CD8α—a population that did not arise with FLT3L treatment alone^49^. By bulk RNA-seq, the transcriptome of sorted Notch-differentiated cDC1s clustered closely with primary cDC1s sorted from the spleen, indicating better recapitulation of physiological cDC1s. Notably, this phenotype translated to improved vaccine efficacy, as vaccination with ovalbumin (OVA)-pulsed Notch-differentiated cDC1s resulted in better survival compared to FLT3L-differentiated cDC1s after challenge with a B16 tumour expressing OVA. This was attributed to enhanced lymph node migration based on upregulation of *Ccr7* and improved in vitro migration toward CCR7 ligands, although increased migration to lymph nodes was not addressed in vivo. Together, these results support the implementation of Notch-based differentiation to generate cDC1s for translational purposes.

In the context of human cells, the phenotype of cDC1s derived from CD34^+^ HSPCs well represents natural cDC1s, with cytokine cocktails containing FLT3L and various other growth factors, e.g., FLT3L, stem cell factor (SCF), GM-CSF, and IL-4 (Ref. ^13^), yielding a small population of *bona fide* CD141^+^CLEC9A^+^XCR1^+^ cDC1s^13,47^. However, severely limited output presents a significant challenge for clinical translation, given the limited amount of starting material and the potential need for high or repeated vaccine doses in a therapeutic setting. In two studies, co-culture of human HSPCs and OP9-DL1 reportedly increased cDC1 yield by as much as 20-fold compared to conventional methods, ranging from ∼4.4 (ref. ^49^) to 11 (ref. ^52^) cDC1s per input cell, depending on the tissue source (peripheral blood^49,52^ vs. cord blood^52^), cytokine cocktail (FLT3L, SCF, and GM-CSF^49^ vs. FLT3L, thrombopoietin, IL-7, and GM-CSF^52^), cell culture conditions (2-week differentiation^49^ vs. 1-week expansion and 3-week differentiation^52^), and stromal cell composition (OP9-DL1 only vs. mixture of OP9 and OP9-DL1^52^). Neither study reported thorough optimization of these parameters, suggesting that yields could likely be improved further. To this end, several important insights should be noted: (i) the inclusion of GM-CSF in the cytokine cocktail is important for maximizing cDC1 enrichment^52^, consistent with previous reports^46^; (ii) the presence of OP9 stromal cells lacking DL1 expression improves pDC yield but limits cDC1 yield in the same culture, while OP9-DL1 alone inhibits pDC development and enriches cDC1s^49,52^, suggesting that the ratio of OP9 to OP9-DL1 could be altered to achieve a defined ratio of pDCs and cDC1s; and (iii) cDC1 yield from peripheral blood monocytes is slightly reduced compared to cord blood^52^, an important limitation to consider in a therapeutic setting in which autologous cells are desirable. Phenotypically, the resulting cDC1s expressed transcriptomes that overlapped strongly with primary cDC1s by scRNA-seq^52^ and NanoString nCounter analysis^49^; functionally, they exhibited normal cytokine responses to toll like receptor (TLR) agonists, including secretion of IL-12, tumour necrosis factor (TNF), and IFN-γ^49,52^; efficiently induced CD4^+^ and CD8^+^ T cell proliferation^49^; and migrated toward XCR1, CCR2, CCR5, and CCR7 ligands. In summary, these studies provide a basis for a highly translatable platform for scaling up the generation of bona fide cDC1s. Further optimization of these protocols, as well as additional functional characterization of these cells, is warranted.

### Genetic reprogramming

The reprogramming of somatic cells via induced expression of key developmental factors is an interesting alternative approach to generate cDC1s. Precedence exists for this type of approach in a MoDC context, with the so-called “SmartDC” platform that utilizes a tricistronic lentiviral vector (LV) encoding for GM-CSF, IL-4, and a melanoma TAA—tyrosine-related protein 2 (TRP2)—to program patient-derived CD14^+^ monocytes to self-differentiate into TRP2-presenting MoDCs^53^. In theory, a similar approach involving LV-induced FLT3L expression should induce self-differentiation of HSPCs into pDCs and cDCs, with the option of incorporating Notch signalling to enrich for cDC1s. As an alternative to cytokine signalling, LV-induced expression of key transcription factors in cDC1 development, namely PU.1, IRF8, and BATF3, was recently reported to successfully reprogram fibroblasts into cDC1-like cells, termed “induced DCs” (iDCs) (ref. ^54^). Derived initially from murine embryonic fibroblasts, these iDCs adopted DC morphology; expressed MHCI/II, XCR1, and CD103; clustered closely with primary splenic cDC1s by scRNA-seq; exhibited an appropriate mature phenotype upon TLR stimulation, including upregulation of CD40 and CD86, as well as secretion of IL-12; and demonstrated the capacity to capture, process, and cross-present exogenous antigens to T cells. A tricistronic vector encoding all three transcription factors in the same cassette, particularly with *Spi1* first in the cassette to maintain high levels of PU.1, maximized programming efficiency and allowed for reprogramming of human adult dermal fibroblasts to CD141^+^CLEC9A^+^ iDCs. Although efficiency was limited—only 0.2% of input cells expressed the APC marker human leukocyte antigen-DR isotype (HLA-DR) at the day 9 endpoint—and further characterization is necessary in human cells, the therapeutic relevance of this type of method is clear, given the ready accessibility of dermal fibroblasts as a starting material.

### Isolation of primary DCs from peripheral blood

Considering the difficulties in generating bona fide cDC1s ex vivo, another option that has been explored is the isolation of primary DCs from apheresis products. However, this is limited by the poor availability of circulating DCs in blood, which represent less than 1.0% of peripheral blood monocytes^4,6^, of which cDC1s are generally the rarest^1^. Notably, reductions in the numbers of circulating DCs have also been detected in melanoma and breast cancer patients^55,56^, and disruptions in cDC1-specific development have been observed in pancreatic and breast cancer models^57^, which could further reduce potential yield. FLT3L mobilization could improve the frequency of cDC1s in the blood^58,59^, but the lack of isolation kits specific to human cDC1s remains a barrier^6^. Reflecting these many obstacles, only primary pDCs^44^ and cDC2s^37,60^ have been tested in the clinic, while primary cDC1 vaccine development is in its infancy. Although recently published preclinical mouse data is promising in terms of efficacy^59^, vaccination with circulating, primary cDC1s remains in pilot clinical studies as part of the collaborative European project, “Professional cross-priming for ovarian and prostate cancer” (PROCROP). It should also be noted that functional deficiencies have been demonstrated in circulating DCs of patients, including decreased cross-presentation capacity and IL-12 secretion^56,61^, which could limit the therapeutic capacity of vaccines derived from circulating DCs.

## Vaccine design: optimizing function

**Fig. 1:**
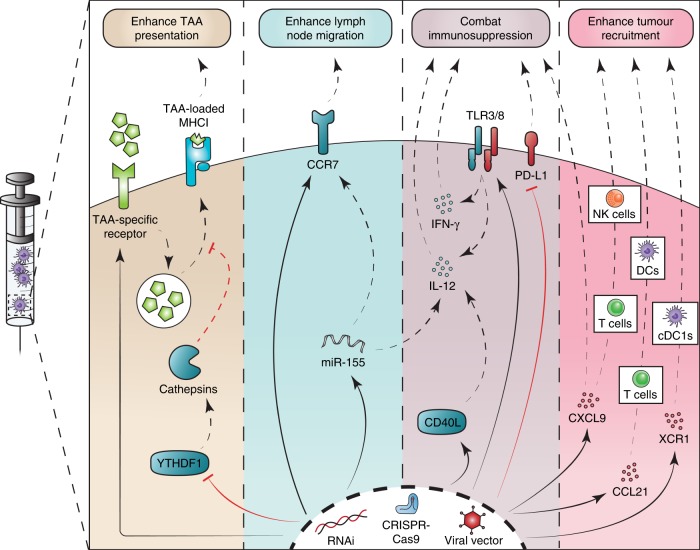
Genetic approaches for improving dendritic cell-based vaccines. RNA interference (RNAi), clustered regularly interspaced short palindromic repeats (CRISPR)/CRISPR-associated protein 9 (Cas9), or viral transduction may be used to modulate expression of different targets and modulate various pathways of anti-tumour immunity. Solid arrows indicate direct genetic targets, while dashed arrows indicate downstream effects. (i) Enhancing tumour-associated antigen (TAA) presentation: expression of an engineered receptor (e.g., extracellular vesicle-internalizing receptor, EVIR) promotes in situ antigen uptake and increases presentation of relevant TAAs^71^; silencing of YTHDF1 reduces translation of lysosomal cathepsins, decreasing antigen degradation after internalization and improving cross-presentation capacity^76^. (ii) Enhancing lymph node migration: direct overexpression of CCR7 improves lymph node migration^63,87^; alternatively, overexpression of miR-155 upregulates CCR7 and IL-12 secretion^29,88,90^. (iii) Abating immunosuppression: silencing dendritic cell (DC)-intrinsic immunosuppressive molecules, such as PD-L1, improves T cell activation capacity^79^; upregulation of IL-12, IFN-γ, or CXCL9 improves immune checkpoint blockade^22,27,31^, which can be achieved via direct overexpression of these signals^80,81^, via activation of TLR3 and TLR8, which induces IL-12 and IFN-γ release^49,52,69^, or via CD40 agonism through transgenic expression of CD40L that activates IL-12 release^31,64,68,82^. (iv) Enhancing immune cell recruitment: upregulation of different chemokines at the tumour site drives recruitment of various immune cells, including CXCL9-driven recruitment of CXCR3^+^ T cells and natural killer (NK) cells^24,83^, CCL21-driven recruitment of CCR7^+^ DCs and T cells^91,92^, and XCL1-driven recruitment of XCR1^+^ cDC1s^26^.

The ex vivo manipulation step presents an important opportunity for improving DC function and vaccine efficacy. Gene-editing technologies may provide means for engineering DCs, including through viral transduction, RNA interference (RNAi), and CRISPR/CRISPR-associated protein 9 (Cas9), all of which have been previously utilized on DCs generated ex vivo^20,53,62–65^. New information regarding the contribution of DCs to tumour immunity should identify actionable molecular targets for optimizing their performance in therapeutic applications. We outline below various strategies for the modification of DCs across a range of anti-tumoural functions (Fig. 1), particularly in the context of cDC1s, providing a toolbox for the rational design of improved DC vaccines.

### Optimizing DC maturation

The importance of using immunologically competent DCs in vaccination applications is well established^66^. Upon stimulation with danger signals, immature DCs upregulate T cell activation machinery, including MHCII, various costimulatory molecules, and immunogenic cytokines. They then migrate to draining lymph nodes, a process driven by CCR7 upregulation. Importantly, tolerance has been observed when vaccinating in the absence of proper maturation factors^66,67^. Various maturation cocktails have been used in the clinic to stimulate MoDCs, generally consisting of different TLR agonists and cytokines, often in combination with co-stimulatory proteins such as CD40 ligand (CD40L). The introduction of endogenously-expressed maturation factors has shown clinical success with the TriMix platform, which involves the introduction of mRNA coding for constitutively-active TLR4, as well as the co-stimulatory proteins CD40L and CD70, via electroporation^68^. This process was shown to induce rapid maturation and survival benefit in melanoma patients. However, ex vivo*-*derived cDC1s and MoDCs respond differently to maturation factors^13^, reflecting the need to re-optimize maturation protocols specifically for cDC1-based vaccines. Notch-differentiated human cDC1s upregulated maturation surface markers after treatment with a range of TLR agonists, but secreted IFN-γ and IL-12 specifically upon TLR3 (poly I:C) and TLR8 (R848) stimulation^49,52^, consistent with increased levels of these cytokines detected in vivo after systemic TLR3/8 activation of humanized mice engrafted with human cDC1s^69^. It has also been demonstrated that activation of TLR3 in CD103^+^ murine cDC1s, which do not respond to TLR8 ligands in contrast to human cDC1s^3^, reverses tumour-driven immunosuppression^30,70^, consistent with the reported role of IFN-γ and IL-12 in cDC1-mediated re-activation of exhausted T cells^31^. These results suggest that TLR3 and TLR8 stimulation should be prioritized for cDC1 maturation, either via cytokine cocktails or genetic engineering, but further characterization is required to confirm these findings in vaccine applications and to extend maturation procedures to a therapeutic scale.

### Enhancing T lymphocyte priming

Given the high efficiency with which cDC1s cross-present antigens to CD8^+^ T cells, replacing MoDCs with cDC1s in vaccines might improve T cell priming. Historically, MoDCs have been loaded ex vivo with specific TAAs by pulsing with peptides, whole proteins, or mRNA to induce endogenous expression and subsequent presentation; however, this requires a priori knowledge of TAAs and is susceptible to acquired resistance via antigen loss. To address these issues, a recent clinical trial used MoDCs pulsed with patient-derived, whole tumour lysate that was oxidized to improve immunogenicity, resulting in efficient loading of a range of TAAs personalized to each patient^12^. This treatment elicited CD8^+^ T cell responses against patient-specific neoantigens, improving progression-free and overall survival in a subset of patients, particularly in combination with immunomodulatory drugs. This approach, although potentially limited to resectable tumours, should be extended to test cDC1-based vaccines.

An interesting alternative could be to leverage antigen loading after vaccine administration, allowing the presentation of patient-specific TAAs in situ, without requiring a biopsy. To this aim we have developed engineered DCs that express a chimeric receptor designed to enhance the uptake of tumour-derived extracellular vesicles (EVs) that display a known surface antigen (called bait antigen). Following uptake, the EV-internalizing receptor (EVIR) allows subsequent presentation of a potentially broad repertoire of TAAs (called prey antigens) that are present inside or on the surface of the EVs^71^. In preliminary mouse studies using HER2 and OVA as model bait and prey antigens, respectively, vaccination with MoDCs expressing a HER2-specific EVIR—without ex vivo antigen loading—led to expansion of OVA-specific T cells and an anti-tumour response. Interestingly, priming of T cells by EVIR-engineered DCs occurred through cross-dressing (Box 1), a process whereby pre-formed MHCI-TAA complexes were horizontally transferred from the tumour-derived EVs to the DCs, as opposed to cross-presentation on endogenous MHCI. Whereas pre-formed, tumour-derived MHCI-antigen complexes may encompass immunologically relevant TAAs, the lack of cross-presentation of EV-associated TAAs might reflect the poor cross-presentation capacity of MoDCs. These observations should encourage exploring the potential of cDC1-based EVIR vaccines.

Improving antigen processing provides another mechanism for enhancing antigen presentation. For example, proteolysis of internalized antigens has been thoroughly demonstrated to regulate cross-presentation^72–76^, providing various targets for intervention. The SNARE protein SEC22B is one option, shown to reduce antigen degradation in DCs by inhibiting recruitment of lysosomal machinery to endosomes^72^. Knockout of SEC22B in CD11c-expressing cells reduced the cross-presentation capacity of DCs and abated the anti-tumour response^75^. However, another study reported contradictory results in the same mouse model^77^, calling for further evaluation of SEC22B as a molecular target for improving cross-presentation. Alternatively, YTHDF1, a protein that promotes the translation of mRNA transcripts marked post-transcriptionally by N^6^-methyadenosine (m^6^A) methylation, was recently shown to upregulate m^6^A-marked lysosomal cathepsins that degrade internalized antigens, thereby decreasing cross-presentation capacity^76^. Knockout of *Ythdf1* in mice improved cross-presentation and delayed growth of B16 and MC38 tumours, without altering DC development, co-stimulatory protein expression, or cytokine secretion. Importantly, improved cross-presentation was observed both in primary splenic cDC1s and FLT3L-differentiated DCs generated ex vivo, which is promising for the extension of this strategy to cDC1-based vaccines using RNAi or CRISPR-Cas9-mediated *YTHDF1* depletion. Although this requires further characterization in human DCs, initial analysis of colon cancer biopsies revealed that low levels of *YTHDF1* correlated with high CD8^+^ T cell abundance in the tumour, indicating the potential relevance of YTHDF1 in human malignancies. Notably, disrupting the m^6^A pathway as a whole was shown to negatively impact DC maturation^78^, which was not detected after specific *Ythdf1* knockout, suggesting that therapeutic intervention downstream of m^6^A methylation is important to retain optimal DC function.

Attenuating tumour-driven suppression of T cells presents another viable therapeutic approach. RNAi and CRISPR-Cas9 technology provide opportunities to silence DC-intrinsic immunosuppressive signals that depress T cell priming. For example, small-interfering RNA (siRNA) knockdown of programmed death-ligand 1 (PD-L1) and PD-L2 in MoDCs improved T cell activation in in vitro studies with human melanoma samples^79^. Notably, bulk gene expression data showed that cDC1s also express PD-L1 and PD-L2 at high levels^6^, suggesting that this strategy could also apply to cDC1-based vaccines. Alternatively, DC-derived signals have been strongly implicated in the ability of immune checkpoint blockade to reinvigorate exhausted T cells in mice^22,23,27,31^, indicating high potential for synergy between these drugs and DC vaccines. For example, the importance of cDC1-derived cytokines IL-12 and CXCL9 for anti-PD-1 and anti-TIM-3 checkpoint blockade has been well demonstrated in multiple studies across different tumour models^22,27,31^. This presents an opportunity to overexpress these cytokines within cDC1-based vaccine formulations, such as through viral transduction of transgenes encoding for IL-12 or CXCL9. Notably, precedence exists for this type of approach in the context of IL-12 (refs. ^80,81^). Manipulating the CD40-CD40L costimulatory axis represents another option, as CD40 agonism induces DC secretion of IL-12 and improves anti-PD-1 treatment^31^. Induction of endogenous CD40L expression via viral^64,82^ or mRNA^68^ delivery is well established as a maturation tool in MoDC vaccines, a technique that could easily be extended to cDC1s for the added synergistic benefit with anti-PD-1 therapy. Overexpression of CXCL9 in cDC1s—perhaps in combination with another canonical CXCR3-binding chemoattractant, CXCL10—could also elicit multiple beneficial effects, with cDC1-derived CXCL9/10 driving effector T cell infiltration in some tumour models^24^. Tumour infiltration by natural killer (NK) cells was shown to also depend on CXCR3 ligands^83^, which may be particularly relevant in the context of cDC1s, as crosstalk between intra-tumoural NK cells and cDC1s has been directly implicated in tumour-driven immunosuppression^26,84^.

### Improving DC migration and trafficking

The abundance of cDC1s in the tumour^24,26,84^ and their ability to deliver TAAs to lymph nodes^28,30^ are both strongly associated with anti-tumour response. Although an ideal vaccine would maximize both lymph node migration and tumour homing, practical and biological limitations will likely impose preferential engineering of one over the other, dependent on the tumour type, its location, and the route of DC administration. Further studies are required to elucidate the impact of the route of administration on DC immunogenicity and vaccine efficacy. Whereas intra-nodal administration of MoDCs significantly increased their abundance in the lymph nodes of melanoma patients compared to intra-dermal administration, no difference in immune response was observed^85^. Similarly, intra-tumoral and intra-nodal administration induced equivalent immune response and efficacy in MoDC-treated breast cancer patients^86^.

Although it remains unclear which administration route should be prioritized moving forward, the inherent trafficking capacity of DCs can be leveraged and optimized, regardless of route. The main strategies to improve lymph node migration focus on the upregulation of CCR7 through a variety of approaches. This was demonstrated with adenoviral transduction of MoDCs generated ex vivo; CCR7 over-expression improved their accumulation in tumour-draining lymph nodes of mice by more than 5-fold after intra-dermal administration^63^. Micelles have also been used to efficiently target CCR7 plasmid DNA to DCs in vivo, promoting migration of skin DCs to lymph nodes and eliciting an anti-tumour response against a B16-OVA model^87^. High transfection efficiency was shown in ex vivo*-*generated MoDCs, supporting the feasibility of non-viral gene delivery for cell therapy applications. Epigenetic regulation of CCR7 provides another opportunity for intervention. Deletion of microRNA-155 (miR-155) in *miR-155*^*–/–*^ BM-derived MoDCs increased trimethylation of histone 3 lysine 27 (H3K27me3), leading to transcriptional repression of CCR7 (refs. ^29,^^88^). Accordingly, overexpression of miR-155, e.g., via retroviral transduction^89^, could elicit multiple beneficial effects in addition to CCR7 overexpression, including DC maturation and IL-12 secretion^29,90^. However, the role of miR-155 has yet to be studied in cDC1s, warranting further investigation. Given the demonstrated importance of CCR7 in cDC1s^28^, the translation of the aforementioned approaches to cDC1-based vaccines should be pursued thoroughly.

Because multiple chemokines are involved in the recruitment of DCs to the tumour, they could be leveraged to drive tumour infiltration of circulating cDC1s and amplify therapeutic responses in situ. Adenoviral-induced expression of chemokine (C-C motif) ligand 21 (CCL21), a CCR7 ligand that also engages T cells, in ex vivo-derived MoDCs improved tumour infiltration of CD4^+^ and CD8^+^ T cells, as well as DEC205^+^ cDC1s, upon intra-tumoural administration in mice^91^. This treatment strategy was tested in a recent phase I clinical trial for NSCLC in which a subset of patients experienced systemic antigen-specific immune responses, increased tumour infiltration by CD8^+^ T cells, and increased PD-L1 expression in the tumour, suggesting the potential for synergy with anti-PD-1/PD-L1 therapy^92^. It was also reported that the chemokines CCL5 and XCL1, which are produced by tumour-resident NK cells, are important for the recruitment of cDC1s and required for anti-tumour immunity^26^. Overexpression of XCL1, a chemokine that engages XCR1 (a receptor specific to cDC1s, as opposed to CCL5 that also recruits pro-tumoural subsets of macrophages and T_regs_), may be used to encourage the specific infiltration of cDC1s into the tumour.

## Outlook

### Vaccine manufacturing: optimizing ex vivo differentiation

**Fig. 2:**
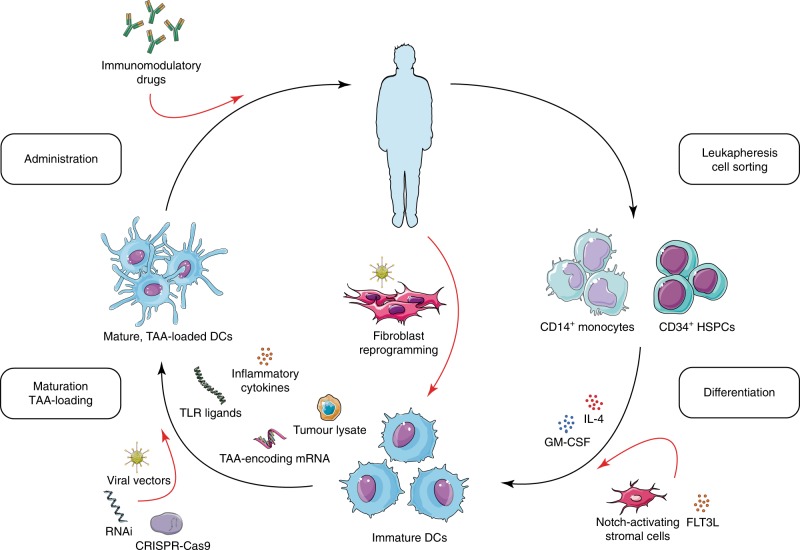
Proposed manufacturing procedures for improving dendritic cell-based vaccines. Black arrows indicate conventional manufacturing steps, while red arrows indicate proposed modifications or additions. CD14^+^ monocytes or CD34^+^ haematopoetic stem and progenitor cells (HSPCs) are isolated from leukapheresis products, differentiated into immature monocyte-derived dendritic cells (MoDCs) with IL-4 and GM-CSF. Alternatively, adult dermal fibroblasts are directly reprogrammed to conventional type 1 DCs (cDC1s) by lentiviral vector (LV) transduction of transcription factors without the need for leukapheresis^54^, or CD34^+^ HSPCs differentiated under FLT3L and Notch signalling to produce cDC1s at high yields^49,52^. The resulting DCs are then loaded with tumour-associated antigens (TAAs) of various forms and matured with toll like receptor (TLR) ligands or inflammatory cytokines. At this step, DCs could be genetically engineered to improve their anti-tumoural functions (see Fig. 1). Mature, TAA-loaded DCs are then re-infused to the patient, which could be combined with immunomodulatory drugs such as immune checkpoint blockade.

DCs—particularly cDC1s—comprise a versatile cell type capable of engaging multiple facets of the immune system, providing a highly modular vaccine platform potentially applicable to a broad range of cancers. However, DC vaccines have yet to be fully realized as an immunotherapy. Although genetically engineered cell therapies have demonstrated remarkable clinical success, such as T cells engineered to express chimeric antigen receptors against B cell malignancies^93^, few clinically tested DC vaccines employ cells that have been modified to optimize function. Based on newly described mechanisms of cDC1-mediated anti-tumour immunity, we propose engineering strategies for manufacturing improved cDC1-based vaccines (Fig. 2). However, the entirety of these modifications cannot be incorporated into a single vaccine; instead, as biomarkers for patient-specific tumour susceptibility improve, we envision the rational design of each vaccine on a patient-to-patient basis, leveraging the flexibility of cDC1s toward a truly personalized treatment strategy.

Co-administration of DC vaccines with other drugs must necessarily be explored for improving efficacy. Potential examples include combination with immune checkpoint blockade, which is already being tested in clinical trials^6^; colony-stimulating factor-1 receptor (CSF1R) blockade-mediated depletion of TAMs^94,95^, which have been shown to directly suppress DC function^96^; neutralization of vascular endothelial growth factor A (VEGFA), which has been shown to impair endogenous DC maturation and function^97^; and therapies that induce the release of TAAs via immunogenic cell death, such as certain chemotherapies^98^ or oncolytic viruses^99^. Future studies should continue to optimize DC vaccines in order to maximize their therapeutic potential.
